# Fixational Eye Movement Waveforms in Amblyopia: Characteristics of Fast and Slow Eye Movements

**DOI:** 10.16910/jemr.12.6.9

**Published:** 2019-07-05

**Authors:** Sarah L. Kang, Sinem B. Beylergil, Jorge Otero-Millan, Aasef G. Shaikh, Fatema F. Ghasia

**Affiliations:** Case Western Reserve University School of Medicine, Cleveland, USA; Biomedical Engineering, Case Western Reserve University, Cleveland, USA; Daroff-Dell’Osso Ocular Motility Laboratory, Louis Stokes Cleveland VA Medical Center, Cleveland, USA; Johns Hopkins University, Baltimore, USA; Department of Neurology, Case Western Reserve University, Cleveland, USA; Neurology service, Louis Stokes Cleveland VA Medical Center, Cleveland, USA; Vision Neurosciences and Ocular Motility Lab, Cole Eye Institute, Cleveland Clinic, Cleveland, USA

**Keywords:** Eye movement, saccades, microsaccades, fixational stability, binocular viewing

## Abstract

Fixational eye movements comprise of fast microsaccades alternating with slow intersaccadic drifts. These physiologic eye movements play an important role in visual perception. Amblyopic patients are known to have fixation instability, particularly of the amblyopic eye. We examined eye movement abnormalities that contribute to this instability. We found that fixation stability is affected by the presence of fusion maldevelopment nystagmus (FMN). However, some amblyopes can have nystagmus without nasally directed slow phases and reversal in direction of the quick phase on ocular occlusion, features seen in FMN. In patients without nystagmus, we found increased amplitude of fixational saccades and inter-saccadic drifts. We categorized amblyopia patients by type (anisometropic, strabismic, or mixed) and eye movement waveform (no nystagmus, nystagmus without FMN, and FMN). We found specific fast and slow eye movement abnormalities of the fellow and amblyopic eye during fellow, amblyopic and both eyes viewing conditions across eye movement waveforms and types of amblyopia. These eye movement abnormalities can serve as biomarkers that can predict the impact of amblyopia as measured by visual acuity and stereopsis. Evaluation of fixational eye movements in amblyopia could be important to diagnose these common eye diseases and predict treatment effectiveness.

## Introduction

The postnatal experience is critical to the continuing development of the visual system. If correlated activity between the two eyes is disrupted during critical developmental periods, amblyopia can result, in which one eye has worse visual acuity that cannot be attributed to any structural abnormality and cannot be corrected by corrective lenses alone ( 1, 2, 3 ). Amblyopia is the most common cause of blindness in children ( 4, 5, 6 ). Amblyopia can arise due to a difference in refractive error between the two eyes (anisometropia), eye misalignment (strabismus), or a mixed mechanism (presence of both anisometropia and strabismus). Amblyopia causes changes in organization and function at the level of the primary visual cortex (V1), with under-representation of the ocular dominance columns corresponding to the amblyopic eye and loss of binocular horizontal cells between ocular dominance columns of opposite ocularity in area V1 ( 2 , 7, 8, 9 ). It is also known that amblyopic patients who have experienced a disruption in binocularity in the first six months of life develop fusion maldevelopment nystagmus (FMN), formerly called latent nystagmus, and nasotemporal (NT) pursuit asymmetry ( 3 , 10, 11, 12, 13, 14, 15 ). Sophisticated psychophysical and visual exploration studies in amblyopia patients have shown that amblyopia impacts the function of both the amblyopic and fellow eyes and is now increasingly being recognized as a disorder of binocular vision, in which both eyes are affected, not just the amblyopic eye ( 16, 17, 18 ). Obtaining reliable measurements to quantify the dysfunction of the fellow and amblyopic eye using complex psychophysical tasks can be daunting in children. The advent of remote video-based eye trackers has allowed for reliable and accurate eye movement assessment in children. The purpose of this paper is to systematically assess the fixation eye movement traces in amblyopia patients and categorize amblyopia patients based on the eye movement waveform characteristics obtained under monocular and binocular viewing conditions. Our overarching goal is to identify oculomotor disease biomarkers that are reflective of the severity and type of amblyopia as well as the binocular function impairment seen in amblyopic patients.

During fixation, when the highest level of visual acuity is achieved, the eyes are not motionless but instead show miniature involuntary fixation movements such as micro-saccades or fixational saccades, inter-saccadic drift, and tremor. Microsaccades induce neuronal modulation in cortical area V1 and in the extra-striate cortex. Microsaccades have been shown to play an important role in visual perception by thwarting neural adaptation and preventing visual fading ( 19, 20, 21, 22 ). Thus, the study of fixational eye movements in amblyopia represents a unique opportunity in understanding their role in abnormal visual processing states. A few studies have evaluated fixational saccades in amblyopia patients and have shown that microsaccades are less frequent and have greater amplitude in the amblyopic eye ( 23 , 24 ). Also, the increase in amplitude of fixational saccades correlates with the severity of amblyopia ( 23 ). Thus, although small physiological microsaccades are known to reduce the effects of Troxler fading, the increased fixational saccade amplitude seen in amblyopia can have unfavorable effects on monocular visual function. In addition, amblyopic patients without nystagmus have increase in the inter-saccadic drift during visual fixation in both the fellow and amblyopic eye ( 13 , 23 , 25 ). Instability of fixation has been reported in amblyopia patients ( 23 , 26 , 27 ). The increased amplitude of fixational saccade, increase inter-saccadic drift and nystagmus can all contribute to increased monocular fixation instability in patients with amblyopia.

Fixational instability has been quantified in most studies to date using the bivariate contour ellipse (BCEA), which is a metric that measures the area over which eye position is dispersed during fixation ( 17 ). BCEA as a measure has several limitations including assumption of normality of the underlying position distributions; thus, the values can be affected by the presence of outliers ( 28 ). Because BCEA is a measure of dispersion of eye position, it takes into account both the fast and slow eye movements and does not identify the presence of FMN ( 29 ). FMN is a characteristic oculomotor deficit suggestive of disruption of binocularity in the first six months of life. Thus, FMN serves as a marker that the amblyogenic risk factors were likely present in early infancy. To identify FMN, eye movements traces have to be systematically analyzed for the direction of fast and slow phase under monocular and binocular viewing conditions ( 3 , 11 ).

The presence of FMN can have implications in terms of monocular and binocular visual function deficits and on treatment effectiveness ( 30 ). Thus, to analyze fixation eye movements in amblyopia patients, it is important to evaluate the waveforms for the presence of nystagmus. Since BCEA is a dispersion measure of eye position, it depicts the spread of eye position around the fixation point but does not reflect how fast the eyes are moving. In other words, it does not take into account the eye velocity during fixation, which is equally important in determining the impact of fixation instability on visual functions ( 16 , 31 , 32 ). We specifically hypothesize that eye movement parameters, namely fixational saccade amplitude and inter-saccadic drift in patients without nystagmus, and quick and slow phase in patients with nystagmus, will be better reflective of the type and severity of amblyopia and binocular function impairment rather than measures of BCEA alone.

Increased fixation instability is also seen in amblyopic patients during binocular viewing. This increased instability during binocular viewing is thought to reflect abnormalities in the vergence pathways ( 27 , 33, 34, 35, 36 ). Vergence BCEA is similar to BCEA in that it involves calculating the standard deviation of eye movements, but uses the difference in eye position between the two eyes at a given time. Thus, similar to monocular BCEA, we hypothesize that vergence BCEA does not provide information about presence of nystagmus and difference of eye velocities between the two eyes, measures that would significantly predict the extent of binocular function impairment in amblyopia. In the presence of normal binocular function, the fixation disparity (the difference between the right and left eye alignment) arising due to the motion of the eyes during fixation stays below a critical threshold thus facilitating binocular fusion ( 37 ). In other words, in healthy subjects the microsaccades occur in the two eyes at the same time with similar amplitudes and direction and have a very small degree of disconjugacy (difference in amplitude between the two eyes) that does not impede binocular fusion ( 38 ). We have found that fixational saccade amplitude of the amblyopic eye was greater in patients with absent stereopsis as compared to patients with gross stereopsis, suggesting that patients with amblyopia have impaired binocular coordination ( 23 ). We have also found that the strabismic patients without amblyopia have increased disconjugacy of fixational saccades ( 34 ). Less is known about the effects of presence of microstrabismus, binocular functions, and severity of amblyopia on the disconjugacy of fixational saccades in amblyopic patients without nystagmus and differences in the quick phase amplitude between the two eyes in patients with nystagmus.

We will first characterize monocular and binocular fixational instability by analyzing eye movement waveforms of fellow and amblyopic eye and compare the BCEA values across eye movement waveforms. We will then quantify several eye movement parameters, namely fixational saccade amplitude and inter-saccadic drift in amblyopia patients without nystagmus and slow phase velocities in patients with nystagmus. We will also compute the disconjugacy between fixational saccades in patients without nystagmus and disconjugacy of quick phases in patients with nystagmus. We hypothesize that the eye position and eye velocity information obtained from eye movement waveform analysis will be better reflective of fellow eye and amblyopic eye instability rather than using the global measure of BCEA alone. We will also examine the effects of amblyopia type, severity and presence of stereopsis on eye movement dynamics across eye movement waveforms. We hypothesize that the fixation instability will systematically increase per the eye movement waveforms irrespective of the severity of amblyopia. We also hypothesize that eye movement waveforms and dynamic eye movement properties would be a better predictor of amblyopia type, severity and stereopsis function than BCEA alone.

## Methods

We recruited 64 subjects (20 controls and 44 amblyopes). The subjects were also grouped based on type of amblyopia (anisometropic = 19, mixed = 18, strabismic = 7) and severity of amblyopia (mild = 21, moderate = 11, severe = 12). There was no significant difference in age between amblyopes and controls (p=0.23, t-test), or between subjects based on presence/absence of nystagmus (p=0.61, one-way ANOVA), severity (p=0.07, one-way ANOVA), or type of amblyopia (p=0.18, one-way ANOVA). Clinical characteristics of each subject are described in supplemental table 1. The subjects did not have any other structural abnormality of the eye or any neurologic disorders. The experimental protocol was approved by the Cleveland Clinic institutional review board and written informed consent was obtained from each participant or parent/legal guardian in accordance with the Declaration of Helsinki.

### Eye Movement Recording

Eye movements were measured using the EyeLink 1000 (SR Research, Ontario, Canada), a noninvasive, high-resolution video-oculography tracker that has a spatial resolution of 0.01° and a temporal resolution of 500 Hz. Subjects wore their corrective lenses if applicable for the experiments to achieve their best-corrected vision. Subjects were seated comfortably in a dark room and the subject’s head was stabilized on a chin rest and forehead support, 55cm away from an LCD computer monitor where the visual stimuli were displayed. The resolution of the monitor was 1024x768 and the monitor was 35cm by 27cm. Eye movements were recorded under monocular viewing conditions, both fellow eye viewing (FEV) and amblyopic eye viewing (AEV) and then binocular viewing conditions. In healthy controls, the right eye was labeled as the fellow eye and the left eye was labeled as the amblyopic eye for the purpose of running eye movement analyses. For monocular viewing conditions, an infrared filter was used to cover the eye, which allows infrared rays to measure eye position but blocks the subject from seeing visible light. Monocular calibration and validation of the right and left eye were done under monocular viewing conditions per the manufacturer’s guidelines. Binocular eye positions were measured during both eye viewing and monocular (fellow and amblyopic eye viewing) conditions.

Subjects were instructed to fixate their gaze on a red circular visual target on the screen with a white background (luminance 144 cd/m ^2^ ) for 45 seconds. The target diameter subtended a 0.5° visual angle.

### Data Analysis

The eye position data was then subject to further analysis. All analyses were performed in Matlab (Mathworks, Natick, MA, USA), GraphPad Prism 7 (La Jolla, CA, USA), and SPSS (IBM, Armonk, NY, USA). Blinks and partial blinks were identified and removed. Blinks were defined as portions of raw data where the pupil information was missing, and partial blinks were defined as portions of data where there was a sudden change in pupil size >50 units/sample. In addition, 100 units (200 milliseconds) of data before and after each blink and partial blink was removed to account for periods when the pupil may have been partially occluded by the eyelid. The characteristics of fixation eye movements were analyzed for all study participants.

The fixation stability was quantified by calculating the bivariate contour ellipse (BCEA) using the following equation:

**(1):**
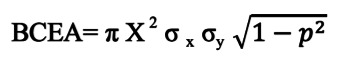


where 2.291 is the Χ ^2^ value (2 degrees of freedom) corresponding to a probability of 0.68, σ _x_ and σ _y_ are the horizontal and vertical standard deviations of eye position respectively, and *p* is the product moment correlation of two position components. The resulting BCEA value has been used in previous studies ( 26 , 27 , 39 ) as a measure of fixation stability, where a lower BCEA indicates more stable fixation. A log _10_ transformation was used to normalize the BCEA values. Vergence BCEA values were also calculated for subjects with binocular viewing data. In vergence BCEA, the standard deviation is taken of the disconjugacy, which is the absolute difference between the viewing and non-viewing eye position in the horizontal and vertical direction.

Fixational saccades and quick phases of nystagmus were identified using the unsupervised clustering method described by Otero-Millan et al. ( 40 ). This method uses clustering to automatically distinguish fixational saccades/quick phases from noise, rather than relying on an arbitrary cutoff. It also produces an index of reliability, which provides a measure of the signal-to-noise ratio in the data. The index of reliability is a number between 0 and 1 and a value greater than 0.75 indicates that error rates are below 0.3 errors per second. Subjects with an index of reliability <0.75 were excluded. Saccade amplitude was defined as the absolute difference between the eye positions at the start and end of a fixational saccade in patients without nystagmus, or quick phase in patients with nystagmus. The horizontal and vertical eye amplitude of the viewing and non-viewing eye were measured and used to calculate a composite amplitude for each eye using the following equation:

**(2):**
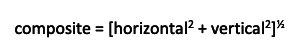


The disconjugacy (difference) of composite amplitude between the viewing and non-viewing eye were computed during fellow, amblyopic and binocular viewing conditions.

Drift analysis was also performed using custom-prepared scripts in Matlab. Drifts were defined as epochs between fixational saccades and blinks in patients without nystagmus, or slow phase velocity in patients with nystagmus. We removed 20 milliseconds of data from the start and end of each of these epochs to exclude periods of acceleration and deceleration of the eye during fixational saccades and blinks. Using horizontal and vertical eye velocity data, the composite mean velocity and composite variability of eye position was computed for the viewing eye and non-viewing eye using the equation given above. The composite mean velocity was compared between controls and groups of amblyopes using one-way ANOVA and planned contrasts. Correlation was also calculated between composite mean velocity and the amplitude of the subsequent microsaccade of the corresponding eye to see if there was a relationship between drift velocity and amplitude of the subsequent microsaccade.

A series of ANOVA analyses were carried out to compare BCEA and eye movement parameters among controls and amblyopic subjects, and between amblyopic subjects with different type and nystagmus waveform characteristics. Visual acuity and stereopsis were included as covariates in all these analyses. We first did a mixed ANOVA to compare BCEA values between controls and amblyopic subjects under fellow and amblyopic eye viewing conditions. Next, a two-way between-subjects ANCOVA was done to compare BCEA values between controls and different types of amblyopia and eye movement waveforms of amblyopic patients. BCEA was the dependent variable and type and waveform were independent variables. Controls were included as a control level of waveform and type. Planned comparisons (Helmert contrasts) were used to compare controls versus amblyopic patients grouped per fixation eye movement waveforms and per the type of amblyopia.

Next, we analyzed fixational saccade amplitude, disconjugacy of amplitude of fixational saccade/quick phase of nystagmus, eye position variance and drift velocity. We performed a series of two-way between-subjects ANCOVA with each of these parameters as the dependent variable in turn, and type and waveform as the independent variables separately for the fellow eye and amblyopic eye. We also included control subjects in this analysis as a control level of waveform and type. We used stereopsis and acuity of the corresponding fellow eye and amblyopic eye as covariates. We used Helmert contrasts to compare controls versus amblyopic patients grouped per fixation eye movement waveforms and per the type of amblyopia.

To analyze binocular eye movements, we first performed a linear correlation between vergence BCEA and stereopsis. Next, a two-way between-subjects ANCOVA was performed with vergence BCEA as the dependent variable and type and waveform as the independent variables. Finally, a two-way between-subjects ANCOVA was performed with disconjugacy under binocular viewing conditions as the dependent variable, and type and waveform as the independent variables. Visual acuity and stereopsis were included as covariates in both analyses.

## Results

 Amblyopic patients are known to have fixation instability, particularly of the amblyopic eye. The presence of nystagmus, the amplitude of fixational saccades and inter-saccadic drifts affect the fixation stability. We have previously shown that amblyopic patients without nystagmus have increased amplitude of fixational saccade, which correlates with the severity of amblyopia (23). The fixation instability of the fellow and amblyopic eye would be dependent on various factors including eye movement waveforms, the type and severity of amblyopia and the stereopsis function. We will first characterize the fixation instability in amblyopia patients based on their eye movement waveform. 

### Characteristics of fixational eye movements in amblyopia patients

**Figure 1.**
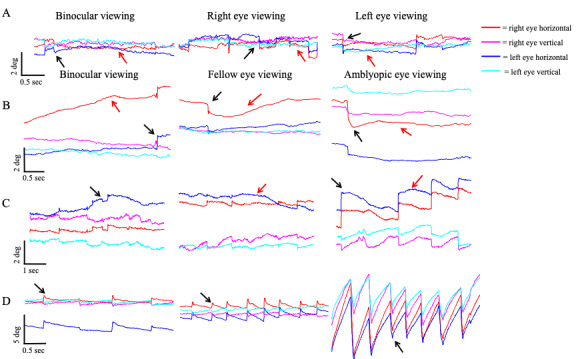
Representative raw data tracings of fixational eye movements under different viewing conditions in a healthy control (N14) (A), severely amblyopic patient without nystagmus (S23) (B), amblyopic patient with nystagmus but no FMN (S30) (C) and a patient with FMN (S44) (D). In the healthy control (A) the horizontal (red and blue) and vertical eye (magenta and cyan) positions of the right and left eye are plotted on the y-axis while x-axis depicts corresponding time. In patients without nystagmus (B) the blue and cyan traces are horizontal and vertical eye positions of the fellow eye and the red and magenta traces are horizontal and vertical eye position of the amblyopic eye respectively. The black arrows represent fixational saccades whereas the red arrows represent the inter-saccadic drifts. In the patient with nystagmus without FMN (C), the red and magenta traces represent horizontal and vertical eye positions of the fellow eye and the blue and cyan represents horizontal and vertical eye positions of the amblyopic eye. The black arrows depict quick phases of the nystagmus and the red arrows depict the slow phases. Fig (D) represents horizontal and vertical eye positions of the fellow (red and magenta) and amblyopic eye (blue and cyan) of patients with fusion maldevelopment nystagmus. The black arrows depict the quick phases of nystagmus. The positive excursion on horizontal eye position = rightward movement and positive excursion on vertical eye position = downward movement.

We characterized fixational eye movements in amblyopic patients based on their waveform characteristics as those without nystagmus and those with nystagmus. Patients with nystagmus were further evaluated for presence of fusion maldevelopment nystagmus (FMN), defined as having a nasally directed slow phase under monocular viewing with classic reversal in the direction of quick phase towards the uncovered eye. We categorized patients with nystagmus who did not meet the criteria of the signature FMN deficits as having nystagmus without FMN. Patients with nystagmus but no FMN did not have the dissociated vertical deviation frequently seen in FMN patients. Fig. 1 contains representative raw data traces of fixational eye movements obtained in a healthy control (A) and amblyopic subjects with no nystagmus (B), nystagmus no FMN (C), and FMN (D) during both eyes viewing, fellow and amblyopic eye viewing conditions. In controls (A) and patients without nystagmus (B) the microsaccades (black arrows) are separated by periods of inter-saccadic drift (red arrows). In the normal subject (N14) the microsaccades are binocular, conjugate movements with small amplitude (< 1°). In patients without nystagmus (S23), there is an increase in the amplitude of the fixational saccades of the amblyopic eye during amblyopic eye viewing condition (2.2°) compared to controls (right eye viewing: 0.74°). The fixation saccade amplitude of the fellow eye is similar to controls (0.92°). Besides the amplitude, there is an increase in the disconjugacy of fixational saccade, which is worse under amblyopic eye viewing compared to fellow eye viewing condition (1.13° vs. 0.20°). Interestingly, the disconjugacy was also increased under both eyes viewing (0.52°). On the other hand, there is minimal disconjugacy in microsaccades during both eyes, right eye and left eye viewing conditions (0.06°, 0.04°, 0.02° respectively). There is also an increase in the inter-saccadic drift velocity and eye position variance of the amblyopic eye (red trace) during both eyes (1.14°/sec, 0.53°) and amblyopic eye viewing (0.4°/sec, 0.08°) viewing conditions compared to controls (both eyes viewing: 0.06°/sec, 0.01°; right eye viewing: 0.3°/sec, 0.01°; left eye viewing: 0.3°/sec, 0.01°). In patients with nystagmus without FMN (S30), the horizontal and vertical eye velocities of the amblyopic eye are greater during amblyopic eye viewing (0.7°/sec and 0.15°) followed by fellow eye viewing (0.6°/sec and 0.01°) conditions. There is no reversal in the direction of the quick phase of the nystagmus between the fellow and amblyopic eye viewing condition. There is an increase in the disconjugacy of the quick phase, which is worst under amblyopic eye viewing (0.41°), but is still increased under fellow eye (0.14°) and both eyes viewing (0.35°) conditions compared to controls. In patients with FMN (S44), the nystagmus is present under all viewing conditions, but the composite eye velocities and eye position variance during slow phases are greater in the amblyopic eye during AEV (19.05°/sec and 3.25°) compared to the fellow eye during FEV condition (4.3°/sec and 0.06°). During both eye viewing condition, the velocity and eye position variance of the amblyopic eye is greater (1.5°/sec and 0.02°) than the fellow eye viewing (0.85°/sec and 0.02°). The slow phases are directed nasally – with rightward movement during the amblyopic left eye viewing condition and the leftward movement during the fellow right eye viewing condition. There is a reversal in the direction of the quick phase of the nystagmus between the fellow and amblyopic eye viewing condition. There is an increase in the disconjugacy during amblyopic eye viewing (0.25°) and fellow eye viewing (0.31°). The disconjugacy is least during both eyes viewing (0.10°) conditions.

 In the subsequent sections, we investigated the effects of type, severity of amblyopia, and binocular function on fixational stability across the eye movement waveforms. We carried out a set of analyses for eye movements obtained under monocular and binocular viewing conditions. We first compared the values obtained using bivariate contour ellipse area (BCEA) analysis between healthy controls and amblyopic subjects. We then examined the correlation of BCEA with amblyopia type and eye movement waveform within amblyopic subjects, while controlling for visual acuity and stereopsis. Next, we repeated this analysis but instead of BCEA we used the composite eye velocity and eye position variance of inter-saccadic drift of controls and patients without nystagmus and composite eye velocity and eye position variance during the slow phases in patients with nystagmus. We also analyzed the composite fixational saccade amplitude and fixational saccade disconjugacy in controls and amblyopic patients without nystagmus. We did a similar analysis of amplitude of quick phase and disconjugacy of amplitude between the two eyes in patients with nystagmus. Lastly, we examined vergence BCEA and disconjugacy as it correlated with amblyopia type and nystagmus waveform in subjects under binocular eye viewing data, while controlling for visual acuity and stereopsis. We used planned contrast analysis to do the following comparisons for waveform characteristics 1) controls versus patients without nystagmus, 2) controls versus patients with nystagmus no FMN, 3) controls versus nystagmus patients with FMN, 4) patients without nystagmus versus patients with nystagmus (with and without FMN), and 5) nystagmus patients without FMN versus nystagmus patients with FMN. Similarly for type of amblyopia, we used planned contrasts for following comparisons 1) controls versus anisometropic amblyopes, 2) controls versus mixed/strabismic, 3) anisometropic versus mixed/strabismic. 

### Bivariate Contour Ellipse Area (BCEA)

**Figure 2.**
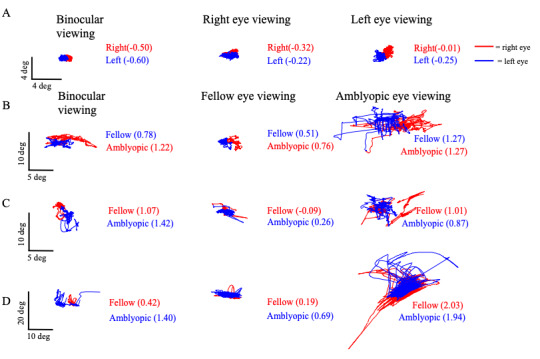
Representative horizontal and vertical eye position data in healthy controls (A) and subjects with amblyopia and no nystagmus (B), nystagmus but no FMN (C), and FMN (D) under fellow, amblyopic, and both eyes viewing conditions. BCEA values (in parentheses) provide a measure of fixational stability, or scatter. The greatest BCEA values are noted under amblyopic eye viewing conditions in both the viewing and non-viewing eye. Interestingly, greater fixational instability is also noted in the amblyopic eye of subjects under binocular viewing. This suggests that fixational stability is affected in amblyopia even when subjects are using both eyes.

Figure 2 plots the horizontal and vertical eye position of controls and subjects with no nystagmus (severe amblyopia), nystagmus no FMN (moderate amblyopia), and FMN (moderate amblyopia) obtained during a 45-second visual fixation trial in primary position during both eyes viewing, fellow eye viewing, and amblyopic eye viewing conditions. These subjects are the same subjects whose eye movement tracings were depicted in Figure 1. The log _10_ BCEA values for both eyes are included next to the scatter plots as a quantitative measure of fixation scatter. As demonstrated in these examples, subjects with amblyopia have greater BCEA values with increased scatter of eye positions particularly during amblyopic eye viewing conditions. We first performed a mixed ANOVA comparing BCEA values in healthy controls and all amblyopic subjects (between-subjects factor) under fellow eye viewing (FEV) and amblyopic eye viewing (AEV) conditions (within-subjects factor). As expected, amblyopic patients had significantly greater BCEA than controls under FEV (controls: -0.41 ± 0.39, amblyopes: 0.32 ± 0.59; F=20.7, p<0.01) and AEV conditions (controls: -0.41 ± 0.38, amblyopes: -0.17 ± 0.38; F=20.7, p<0.01). The difference was more pronounced under AEV as indicated by a significant effect between viewing condition and subject group (F=12.9, p<0.01). These findings are in agreement with previous studies that have shown that amblyopic patients have increased BCEA of the amblyopic eye ( 23 , 26 , 27 ). We also found increased BCEA values of the amblyopic eye during binocular viewing conditions (controls right eye: -0.55 ± 0.23, fellow eye: -0.20 ± 0.55, amblyopic eye: 0.10 ± 0.60; F=8.6, p<0.01).

**Figure 3.**
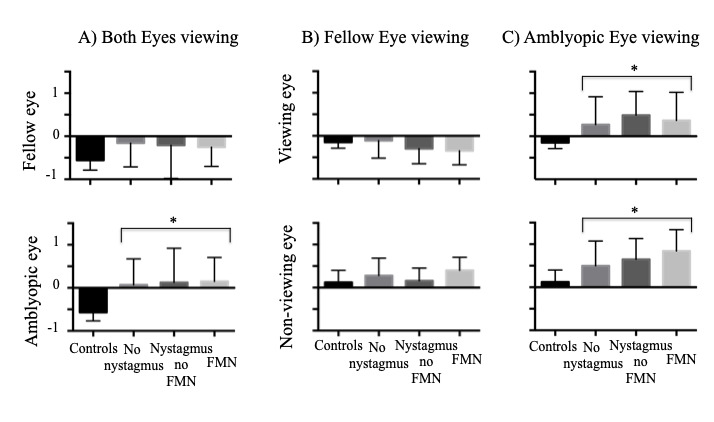
Mean and standard deviation of BCEA values in subjects categorized by fixation eye movement waveforms. Under both eyes viewing condition (A), the amblyopic eye had less stable fixation (positive values) compared to controls (negative values) irrespective of the waveform type. Under fellow eye viewing condition (B), no differences were seen in the viewing and non-viewing eye between controls and amblyopic patients. Under amblyopic eye viewing condition (C), the BCEA values were greater in both the viewing and non-viewing eye. *=p<0.05, one way ANOVA

Next we wanted to discern the effects of waveform and type of amblyopia on BCEA. We looked at BCEA values of controls and amblyopes grouped by eye movement waveform during both eyes viewing (BEV), fellow eye viewing (FEV) and amblyopic eye viewing (AEV) conditions (Figure 3). Under BEV (Fig 3A), the BCEA values were significantly higher in the amblyopic eye compared to controls (controls: -0.57 ± 0.19, no nystagmus: 0.06 ± 0.60, nystagmus no FMN: 0.12 ±0.79, FMN: 0.15 ± 0.55; F=6.6, p<0.01) with no significant difference in the fellow eye (controls: -0.55 ± 0.23, no nystagmus: -0.15 ± 0.55, nystagmus no FMN: -0.20 ± 0.76, FMN: -0.25 ± 0.44; F=2.1, p=0.10). When comparing the BCEA values between groups under FEV (Fig 3B), there was no significant difference between controls and amblyopic patients (controls: -0.15 ± 0.23, no nystagmus: -0.11 ± 0.40, nystagmus no FMN: -0.29 ± 0.35, FMN: -0.34 ± 0.32; F=1.6, p=0.18). In patients with nystagmus, the rhythmic to-and-fro nature of the nystagmus results in densely packed eye positions thus producing less scatter. Thus, during FEV conditions, despite having nystagmus, the BCEA values were actually better for patients with FMN than controls. During AEV (Fig 3C), the BCEA values were higher in amblyopic patients compared to controls (controls: -0.41 ± 0.39, no nystagmus: 0.19 ± 0.59, nystagmus no FMN: 0.46 ± 0.55, FMN: 0.33 ± 0.61; F=9.1; p<0.01). A planned contrast analysis identified differences between controls versus patients without nystagmus, controls versus patients with nystagmus without FMN, controls versus patients with FMN (all Helmert contrasts were significant at p<0.01). No differences were observed between patients without nystagmus versus those with nystagmus and patients without nystagmus without FMN and patients with FMN (p>0.05). Thus, under amblyopic eye viewing similar to fellow eye viewing data, BCEA measures do not reflect the fixation instability occurring due to nystagmus. For the non-viewing eye, significant differences were noted between controls and amblyopia patients across eye movement waveforms under AEV (controls: 0.12 ± 0.27, no nystagmus: 0.49 ± 0.57, nystagmus no FMN: 0.64 ± 0.48, FMN: 0.84 ±0.49; F=5.5, p<0.01). No such differences were seen in the non-viewing eye under FEV (controls: 0.12 ± 0.27, no nystagmus: 0.27 ± 0.40, nystagmus no FMN: 0.15 ± 0.29, FMN: 0.40 ± 0.30; F=1.8, p=0.14).

We also evaluated BCEA values as a function of type of amblyopia during BEV, FEV and AEV conditions. Table 1 summarizes the BCEA values as a function of type of amblyopia. The amblyopic eye had greater BCEA values compared to controls under BEV irrespective of the type of amblyopia. During BEV, the BCEA values of the fellow eye and amblyopic eye in amblyopic patients were worse than controls irrespective of the type of amblyopia. A planned contrast analysis identified differences between controls and fellow eye of anisometropic amblyopia (p=0.04) and controls and patients with mixed/strabismic amblyopia (p=0.01). Similarly, differences were seen between controls and the amblyopic eye of amblyopes during BEV. No differences were observed between anisometropic versus mixed/strabismic amblyopia in the fellow eye (p=0.78) and amblyopic eye (p=0.46).

**Table 1:** BCEA and Amblyopia Type

	Control	Anisometropic	Mixed/Strabismic	Helmert contrast (p-value)
Both eyes viewing	-0.55 ± 0.23* ^ x ^ (-0.57 ± 0.19)* ^ x ^	-0.21 ± 0.47* (0.03 ± 0.63)*	-0.08 ± 0.62 ^ x ^ (0.24 ± 0.57) ^ x ^	0.02 (<0.01)
Fellow Eye viewing	-0.17 ± 0.15 (0.03 ± 0.20)	-0.20 ± 0.29 (0.13 ± 0.30)	-0.22 ± 0.42 (0.35 ± 0.36)	0.9 (0.01)
Amblyopic Eye viewing	-0.17 ± 0.15* ^ x ^ (0.03± 0.20)	0.19 ± 0.56* (0.43 ± 0.40)	0.57 ± 0.71 ^ x ^ (0.77 ± 0.62)	<0.01 (<0.01)

Both eyes viewing: top row: fellow eye, bottom row with parenthesis = amblyopic eye; Fellow and Amblyopic eye viewing: top row: viewing eye, bottom row: non-viewing eye; * = statistically significant values for planned contrast between controls and anisometropic amblyopia; ^x^ =statistically significant values for planned contrast between controls and strabismic/mixed amblyopia

During FEV, the BCEA values of the fellow eye were similar in amblyopic patients compared to controls irrespective of the type of amblyopia. The amblyopic eye had greater BCEA values compared to controls under AEV irrespective of the type of amblyopia. A planned contrast analysis identified differences between controls and amblyopic eye of anisometropic amblyopia (p<0.01), as well as controls and amblyopic eye of patients with mixed/strabismic amblyopia (p<0.01). No differences were observed between anisometropic versus mixed/strabismic amblyopia (p=0.11).

Next, we used a two-way between-subjects ANCOVA to compare BCEA values of the amblyopic eye obtained under amblyopic eye viewing condition across different types of amblyopia (anisometropic and mixed/ strabismic) and nystagmus waveforms (no nystagmus, nystagmus no FMN, and FMN). We also included control subjects in this analysis as a control level of waveform and type. Previous studies have shown that BCEA values are affected by visual acuity and stereopsis ( 23 , 26 , 27 ). Thus, we included visual acuity and stereopsis as covariates to control for these factors. There was no significant effect of waveform and type on BCEA when we controlled for acuity of amblyopic eye and stereopsis (type: F=2.9, p=0.09; waveform: F=2.0, p=0.14). There was a significant effect of the covariate stereopsis (F=12.8, p=0.01) but not the visual acuity (F=0.8, p=0.77).

Fixation instability has been reported in amblyopic patients under both eyes viewing condition ( 17 ). We further calculated a vergence BCEA (right eye position minus left eye position) that assesses fixational stability between the two eyes obtained under both eyes viewing (BEV) conditions. We examined vergence BCEA across fixation waveforms and amblyopia types (Table 2). BCEA values were greater in amblyopic patients compared to controls. A planned contrast analysis identified differences between controls versus patients without nystagmus, controls versus patients with nystagmus no FMN, and controls versus patients with FMN (all Helmert contrasts were significant at p<0.01). No differences were observed between patients without nystagmus versus those with nystagmus and patients without nystagmus no FMN and patients with FMN. A planned contrast analysis identified differences between controls and anisometropic amblyopia patients (p=0.02), as well as controls and mixed/strabismic amblyopia patients (p<0.01). No differences were observed between anisometropic versus mixed/strabismic amblyopia (p=0.27).

**Table 2:** Vergence BCEA

Controls	No nystagmus	Nystagmus no FMN	FMN	Helmert contrast (p-value)
-0.77 ± 0.21	-0.34 ± 0.61	0.09 ± 0.53*	-0.06 ± 0.52*	<0.01
Controls	Anisometropic	Mixed/Strabismic		
-0.77 ± 0.21	-0.31 ± 0.71	-0.15 ± 0.40 ^ x ^		<0.01

* = statistically significant values for planned contrast between controls and amblyopia patients across fixation eye movement waveforms , ^x^ = statistically significant values for planned contrast between controls and strabismic/mixed amblyopia.

We also investigated the relationship between fixational eye movements by using vergence BCEA and stereopsis. A positive correlation was noted between smaller vergence BCEA suggestive of stable fixation and stereopsis function (Spearman’s rho correlation coefficient=0.64, p<0.01).

We used a two-way between-subjects ANCOVA to compare vergence BCEA values between amblyopic subjects, comparing between types of amblyopia (anisometropic, mixed, and strabismic) and nystagmus waveforms (no nystagmus, nystagmus no FMN, and FMN). We also included control subjects in this analysis as a control level of waveform and type. We included visual acuity and stereopsis as covariates to control for these factors. There was no significant effect of waveform and type on BCEA when we controlled for acuity of amblyopic eye and stereopsis (type: F=0.19, p=0.66; waveform: F=0.44, p=0.65).

Thus, BCEA and vergence BCEA are able to distinguish amblyopic patients from controls. However, the BCEA measures are not able to identify differences between amblyopic patients based on clinical type or fixation eye movement waveforms. This highlights the importance of incorporating dynamic eye movement parameters such as eye velocity, eye position variance, fixational saccade amplitude, and disconjugacy that would better reflect the fixation instability during monocular and binocular viewing conditions.

### Comparison of Eye Movement Parameters as a function of type and fixation eye movement waveforms 

We have previously shown that the amplitude of fixational saccades of the amblyopic eye of patients without nystagmus is increased compared to controls ( 23 ). The increase in amplitude is correlated with the severity of amblyopia. Increased eye velocity and variance of eye positions during drifts (epochs between two consecutive fixational saccades) are seen in patients with visual loss ( 41 ). We have previously found an increase in the drift velocity and eye position variance of both the fellow and amblyopic eye of amblyopic patients without nystagmus, which correlate with an increase in amblyopia severity ( 16 , 23 ). On the other hand, although the slow phase velocities in patients with fusion maldevelopment nystagmus are increased, the increase did not correspond to an increase in the severity of amblyopia ( 16 ). Thus, in the current study, we investigated the effects of amblyopia severity and stereopsis function on the composite amplitude of fixational saccades in patients without nystagmus and quick phases in patients with nystagmus during both eyes viewing, fellow eye viewing and amblyopic eye viewing conditions. We did a similar analysis on the inter-saccadic drift velocity in patients without nystagmus and slow phase velocity of patients with nystagmus with and without FMN. We will first describe the results obtained under both eyes viewing condition followed by fellow and amblyopic eye viewing conditions.

### Both eyes viewing condition, Composite Amplitude

**Figure 4.**
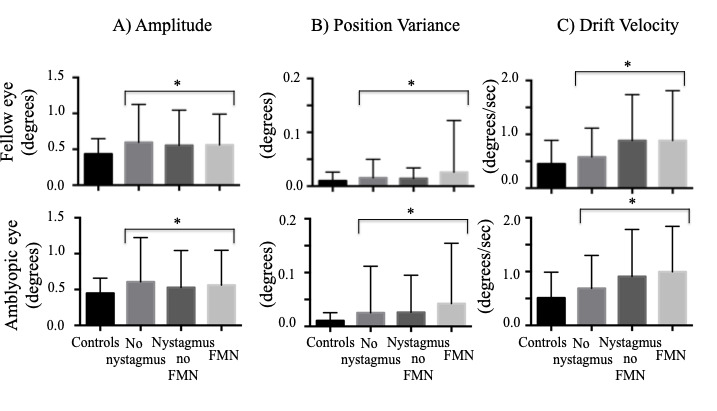
Mean and standard deviation of eye movement parameters of fixational saccade amplitude, position variance, and drift velocity in subjects across eye movement waveforms under both eyes viewing condition. Composite amplitude (A) of fixational saccades in controls and patients without nystagmus and quick phases of patients with nystagmus shows increased amplitude in both the fellow and amblyopic eye of amblyopes. Similarly, position variance (B) of drifts in controls and patients without nystagmus and slow phases of patients with nystagmus is also increased in both the fellow and amblyopic eye, although the increase is more pronounced in the amblyopic eye. The drift velocity (C) in controls and patients without nystagmus and slow phase velocities in patients with nystagmus is also increased in amblyopes compared to controls. Those with and without nystagmus have greater velocity compared to controls. *=p<0.05, one way ANOVA

We found an increase in the amplitude of the fixational saccades of both the fellow and amblyopic eye during both eye viewing conditions. We found a similar increase in the amplitude of the quick phase of patients with nystagmus (Fig 4A). The differences were statistically significant across fixation eye movement waveforms (fellow eye: controls: 0.43° ± 0.21°, no nystagmus: 0.59° ± 0.52°, nystagmus no FMN: 0.55° ± 0.48°, FMN: 0.56° ± 0.42°; F=10.9, p<0.01; amblyopic eye: controls: 0.44° ± 0.20°, no nystagmus: 0.61° ± 0.61°, nystagmus no FMN: 0.52° ± 0.51°, FMN: 0.56° ± 0.48°; F=7.1, p<0.01). A planned contrast analysis identified differences between controls and patients without nystagmus, controls and patients with nystagmus no FMN and control and patients with FMN for fellow and amblyopic eye (Helmert contrasts were significant at p<0.05). No differences were seen between patients without nystagmus and patients with nystagmus, and patients with nystagmus with and without FMN for fellow and amblyopic eye (p>0.05).

We also evaluated composite amplitude as a function of type of amblyopia during both eyes viewing condition. The fellow (controls: 0.43° ± 0.21°, anisometropia: 0.57° ± 0.50°, mixed/strabismic: 0.57° ± 0.47°; p<0.01) and amblyopic eye (controls: 0.44° ± 0.29°, anisometropia: 0.56° ± 0.61°, mixed/strabismic: 0.57° ± 0.52°, p<0.01) had greater amplitude compared to controls irrespective of the type of amblyopia. A planned contrast analysis identified differences between controls and fellow eye of patients with anisometropia (p<0.01) and controls and patients with mixed/strabismic amblyopia (p<0.01). Similarly, differences were seen between controls and amblyopic eye of anisometropic (p<0.01) and mixed/strabismic amblyopia (p<0.01) patients during BEV. No differences were observed between anisometropic versus mixed/strabismic amblyopia in the fellow eye (p=0.95) and amblyopic eye (p=0.43).

A two-way between-subjects ANCOVA was carried out to examine the effects of type and waveform on the amplitude of the fellow and amblyopic eye obtained under both eye viewing. There was a statistically significant main effect of type (F=9.5, p<0.01) and waveform (F=6.3, p<0.01), on amplitude of the fellow eye whilst controlling for visual acuity and stereopsis. In addition, the covariates visual acuity (F=8.4, p<0.01) and stereopsis (F=8.1, p <0.01) did have significant effects on the amplitude of the fellow eye. Similarly, there was a statistically significant main effect of type (F=6.2, p=0.01) and waveform (F=6.6, p<0.01), on amplitude of the amblyopic eye whilst controlling for visual acuity and stereopsis. In addition, the covariates acuity (F=5.9, p=0.01) and stereopsis (F=9.4, p<0.01) did have main effects on the amplitude of the amblyopic eye.

### Eye position variance during inter-saccadic drifts and slow phase velocity

We first compared position variance across eye movement waveforms. We found an increase in the eye position variance of the fellow eye of amblyopic patients under both eyes viewing conditions (controls: 0.01° ± 0.01°, no nystagmus: 0.01° ± 0.03°, nystagmus no FMN: 0.01° ± 0.01°, FMN: 0.02° ± 0.09°; F=6.3, p<0.01). A similar increase was seen in the amblyopic eye across all eye movement waveforms (controls: 0.01° ± 0.01°, no nystagmus: 0.02° ± 0.08°, nystagmus no FMN: 0.02° ± 0.06°, FMN: 0.04° ± 0.11°; F=13.8, p<0.01) (Fig 4B). A planned contrast analysis showed a significant increase in variance in patients without nystagmus compared to controls, patients with nystagmus without FMN compared to controls, patients with FMN compared to controls and FMN patients compared to patients with nystagmus without FMN for both the fellow and amblyopic eye (all Helmert contrasts were significant at p<0.01).

We also evaluated eye position variance as a function of type of amblyopia during both eyes viewing conditions. The fellow eye (controls: 0.01° ± 0.01°, anisometropia: 0.02° ± 0.03°, mixed/strabismic: 0.02° ± 0.07°; F=11.3, p<0.01) and amblyopic eye (controls: 0.01° ± 0.01°, anisometropia: 0.03° ± 0.11°, mixed/strabismic: 0.03° ± 0.08°; F=20.76, p<0.01) had greater eye position variance compared to controls irrespective of the type of amblyopia. A planned contrast analysis identified differences between controls and fellow eye of patients with anisometropia (p<0.01) and controls and patients with mixed/strabismic amblyopia (p<0.01). Similarly, differences were seen between controls and amblyopic eye of anisometropic (p<0.01) and mixed/strabismic amblyopia (p<0.01) patients during BEV. No differences were observed between anisometropic versus mixed/strabismic amblyopia in the fellow eye (p=0.72) and amblyopic eye (p=0.75).

A two-way between-subjects ANCOVA was carried out to examine the effects of type and waveform on the amplitude of the fellow and amblyopic eye obtained under both eye viewing condition while controlling for visual acuity and stereopsis. There was no statistically significant main effect of the type (F=0.27, p=0.6) and waveform (F=0.94, p=0.39) on eye position variance of the fellow eye. On the other hand, for the amblyopic eye there was a statistically significant main effect of the type (F=10.1, p<0.01) and waveform (F=9.5, p<0.01), whilst controlling for visual acuity and stereopsis. The covariate acuity of the amblyopic eye showed a significant effect on eye position variance of the amblyopic eye under both eyes viewing condition (F=8.9, p<0.01).

### Eye velocity during inter-saccadic drifts and slow phase velocity

We also found an increase in the inter-saccadic drift and slow phase velocity of both the fellow and amblyopic eye during both eye viewing conditions across eye movement waveforms (Fig 4C). The differences were statistically significant for both the fellow eye (controls: 0.45°/s ± 0.43°/s, no nystagmus: 0.57°/s ± 0.53°/s, nystagmus no FMN: 0.88°/s ± 0.85°/s, FMN: 0.88°/s ± 0.92°/s; F=30.1, p<0.01) and amblyopic eye (controls: 0.51°/s ± 0.47°/s, no nystagmus: 0.68°/s ± 0.61°/s, nystagmus no FMN: 0.90°/s ± 0.87°/s, FMN: 0.99°/s ± 0.84°/s; F=34.3, p<0.01). A planned contrast analysis identified differences between controls and patients without nystagmus, controls and patients with nystagmus no FMN and control and patients with FMN for fellow and amblyopic eye (Helmert contrasts were significant at p<0.01). The eye velocities were greater in patients with nystagmus compared to patients without nystagmus for fellow and amblyopic eye (p<0.01).

We also evaluated eye velocity as a function of type of amblyopia during both eyes viewing conditions. The fellow (controls: 0.45°/s ± 0.43°/s, anisometropia: 0.80°/s ± 0.84°/s, mixed/strabismic: 0.75°/s ± 0.77°/s; F=39.4, p<0.01) and amblyopic eye (controls: 0.51°/s ± 0.47°/s, anisometropia: 0.80°/s ± 0.85°/s, mixed/strabismic: 0.87°/s ± 0.75°/s; F=46.3, p<0.01) had greater eye velocities compared to controls irrespective of the type of amblyopia. A planned contrast analysis identified differences between controls and fellow eye of patients with anisometropia and controls and patients with mixed/strabismic amblyopia. Similarly, differences were seen between controls and amblyopic eye of anisometropic and mixed/strabismic amblyopia patients during BEV. All Helmert contrasts were significant at p<0.01. No differences were observed between anisometropic versus mixed/strabismic amblyopia in the fellow eye (p=0.43) and amblyopic eye (p=0.37).

A two-way between-subjects ANCOVA was carried out to examine the effects of type and waveform on the eye velocity of the fellow and amblyopic eye obtained under both eye viewing condition while controlling for visual acuity and stereopsis. There was a statistically significant main effect of type (F=13.9, p<0.01) and waveform (F=21.7, p<0.01) on the velocity of the fellow eye. A similar statistically significant main effect of type (F=8.1, p<0.01) and waveform (F=11.0, p<0.01) was seen on the velocity of the amblyopic eye while controlling for visual acuity and stereopsis. In addition, there was a significant effect of covariate stereopsis (F=6.4, p=0.01) but not the visual acuity (F=1.2, p=0.26) on the eye velocity of the amblyopic eye.

### Fellow and amblyopic eye viewing conditions, Composite Amplitude

**Figure 5.**
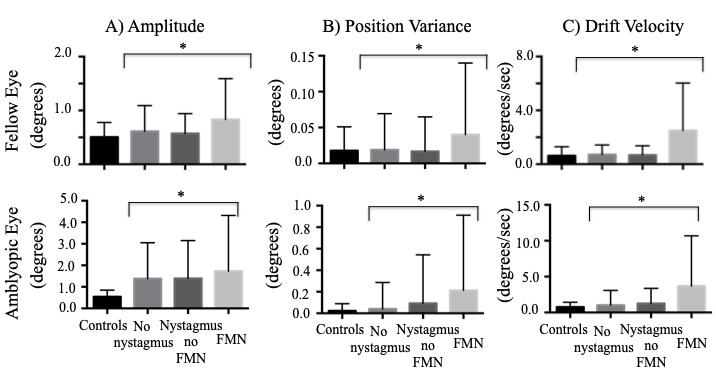
Mean and standard deviation of eye movement parameters of fixational saccade amplitude, position variance, and drift velocity in subjects across eye movement waveforms under fellow and amblyopic eye viewing conditions. Error bars represent one standard deviation. Composite amplitude (A) of fixational saccades in controls and patients without nystagmus and quick phases of patients with nystagmus shows increased amplitude in amblyopes under both fellow and amblyopic eye viewing. Similarly, position variance (B) of drifts in controls and patients without nystagmus and slow phases of patients with nystagmus is also increased. The drift velocity (C) in controls and patients without nystagmus and slow phase velocities in patients with nystagmus is also increased in amblyopes compared to controls. *=p<0.05, one way ANOVA

When comparing the composite amplitude between groups of nystagmus waveforms, the amplitude was greater in amblyopic patients during both fellow eye viewing (controls: 0.51° ± 0.27°, no nystagmus: 0.61° ± 0.47°, nystagmus no FMN: 0.57° ± 0.36°, FMN: 0.83° ± 0.75°; F=58.9, p<0.01) and amblyopic eye viewing conditions (controls: 0.54° ± 0.30°, no nystagmus: 1.30° ± 1.60°, nystagmus no FMN: 1.20° ± 1.20°, FMN: 1.70° ± 2.50°; F=25.1, p<0.01) (Figure 5A). A planned contrast analysis identified differences during both fellow and amblyopic eye viewing conditions between controls versus patients without nystagmus, controls versus patients with nystagmus no FMN, controls versus patients with FMN (Helmert contrasts were significant at p<0.01). Unlike the BCEA, significant differences were observed between patients without nystagmus versus those with nystagmus (p<0.01), and patients without nystagmus no FMN and patients with FMN (p<0.01) during both fellow and amblyopic eye viewing conditions.

When comparing the composite amplitude between different types of amblyopia, the composite amplitude of the fellow eye was greater in amblyopic patients during FEV compared to controls irrespective of the type of amblyopia (controls: 0.50° ± 0.27°, anisometropic: 0.56° ± 0.38°, mixed/strabismic: 0.71° ± 0.62°; F=48.3, p<0.01). A greater increase of composite amplitude of the amblyopic eye was seen during amblyopic eye viewing condition irrespective of the type of amblyopia (controls: 0.54° ± 0.30°, anisometropic: 1.09° ± 1.34°, mixed/strabismic: 1.50° ± 2.10°; F=40.6, p<0.01). A planned contrast analysis identified differences between controls and fellow eye and controls and amblyopic eye of anisometropic amblyopia (p<0.01). Similar differences were seen with planned contrast analysis with greater amplitude of the fellow and amblyopic eye of strabismic/mixed amblyopia patients compared to controls (p<0.01). We also found a greater increase in amplitude of the fellow and amblyopic eye of mixed/strabismic amblyopia patients compared to anisometropia patients (p<0.01, p<0.01).

A two-way between-subjects ANCOVA was carried out to examine the effects of type and waveform on the amplitude of the fellow eye obtained during fellow eye viewing condition. There was a statistically significant main effect of the type (F=75.4, p<0.01) and waveform (F=28.5, p<0.01) on amplitude of the fellow eye whilst controlling for visual acuity and stereopsis. The covariates acuity (F=2.7, p=0.10) and stereopsis (F=0.1, p=0.80) did not have any significant effects on the amplitude of the fellow eye. A similar analysis was done for the amplitude of the amblyopic eye obtained during amblyopic eye viewing condition. There was a statistically significant main effect of the type (F=14.2, p<0.01) and waveform (F=13.06, p<0.01) on amplitude of the amblyopic eye whilst controlling for visual acuity and stereopsis. The covariates acuity (F=28.8, p<0.01) and stereopsis (F=53.3, p<0.01) showed significant effects on the amplitude of the amblyopic eye.

### Eye position variance during inter-saccadic drifts and slow phase velocity

We found an increase in the eye position variance of the fellow eye of amblyopic patients (controls: 0.01° ± 0.03°, no nystagmus: 0.01° ± 0.05°, nystagmus no FMN: 0.01° ± 0.04°, FMN: 0.04° ± 0.09°; F=11.6, p<0.01). A planned contrast analysis showed an increase in variance of slow phases in patients with FMN compared to the other groups (p<0.01). An increase was also seen in amblyopic eye across all fixation eye movement waveforms (controls: 0.01° ± 0.06°, no nystagmus: 0.04° ± 0.25°, nystagmus no FMN: 0.09° ± 0.40°, FMN: 0.21° ± 0.69°; F=23.4, p<0.01) (Figure 5B). A planned contrast analysis identified differences between controls and patients without nystagmus, controls and patients with nystagmus without FMN, controls and patients with FMN, patients with and without nystagmus, and patients with and without FMN (Helmert contrasts were significant at p<0.01).

When comparing the eye position variance between groups of type of amblyopia, the eye position variance of the fellow eye was greater in amblyopic patients during FEV compared to controls (controls: 0.01° ± 0.03°, anisometropic: 0.01° ± 0.02°, mixed/strabismic: 0.02° ± 0.08°; F=24.3, p<0.01). A planned contrast analysis identified differences between controls and fellow eye of patients with mixed/strabismic amblyopia, and patients with anisometropia and those with mixed/strabismic amblyopia (all Helmert contrasts were significant at p<0.01). A greater increase of eye position variance of the amblyopic eye was seen during amblyopic eye viewing condition irrespective of the type of amblyopia (controls: 0.01° ± 0.06°, anisometropic: 0.03° ± 0.10°, mixed/strabismic: 0.14° ± 0.58°; F=33.6, p<0.01). A planned contrast analysis identified differences between controls and amblyopic eye of anisometropic amblyopia (p=0.03) and controls and amblyopic eye of mixed/strabismic amblyopia (p<0.01) and anisometropic and mixed/strabismic amblyopia (p<0.01).

A two-way between-subjects ANCOVA was carried out to examine the effects of type and waveform on the eye position variance of the fellow eye obtained during fellow eye viewing condition. We found a statistically significant main effect of type (F=13.4, p <0.01) and waveform (F=16.7, p<0.01) on eye position variance of the fellow eye (F=65.8, p<0.01) whilst controlling for visual acuity and stereopsis. The covariates acuity (F=9.3, p=0.02) and stereopsis (F=3.7, p=0.05) also had significant effects on the eye position variance of the fellow eye. A similar analysis was done for eye position variance of the amblyopic eye obtained during amblyopic eye viewing condition. There was a statistically significant main effect of the waveform (F=7.4, p<0.01) but not the type of amblyopia (F=2.6, p=0.1) whilst controlling for visual acuity and stereopsis. The covariates stereopsis (F=24.0, p<0.01) but not the visual acuity of the amblyopic eye (F=0.2, p=0.64) had significant effects on the eye position variance of the amblyopic eye.

### Eye velocity during inter-saccadic drifts and slow phase velocity

When comparing the eye velocity between groups of eye movement waveforms, the velocity was greater under both fellow eye viewing (controls: 0.63°/s ± 0.66°/s, no nystagmus: 0.71°/s ± 0.71°/s, nystagmus no FMN: 0.68°/s ± 0.68°/s, FMN: 2.50°/s ± 3.50°/s; F=66.1, p<0.01) and amblyopic eye viewing (controls: 0.76°/s ± 0.65°/s, no nystagmus: 1.03°/s ± 2.0°/s, nystagmus no FMN: 1.20°/s ± 1.80°/s, FMN: 3.60°/s ± 6.90°/s; F=53.0, p<0.01) of amblyopic patients (Figure 5C). A planned contrast analysis identified differences during both fellow and amblyopic eye viewing conditions between controls versus patients without nystagmus (p<0.01) and controls versus patients with FMN (p<0.01). Unlike the BCEA, differences were observed between patients without nystagmus versus those with nystagmus, and patients without nystagmus no FMN and patients with FMN during both fellow and amblyopic eye viewing conditions (all Helmert contrasts were significant at p<0.01).

When comparing the eye velocity between groups of type of amblyopia, the eye velocity of the fellow eye was greater in amblyopic patients during FEV compared to controls irrespective of the type of amblyopia (controls: 0.63°/s ± 0.66°/s, anisometropic: 0.67°/s ± 0.74°/s, mixed/strabismic: 1.50°/s ± 2.50°/s; F=85.4, p<0.01). A greater increase of eye velocity of the amblyopic eye was seen during amblyopic eye viewing condition irrespective of the type of amblyopia (controls: 0.67°/s ± 0.65°/s, anisometropic: 0.84°/s ± 1.06°/s, mixed/strabismic: 2.40°/s ± 5.30°/s; F=78.4, p<0.01). A planned contrast analysis did not identify any difference between controls and anisometropic amblyopia under fellow eye or amblyopic eye viewing condition (p=0.20). However, strabismic/mixed amblyopia patients had significantly higher eye velocity of the fellow and amblyopic eye of compared to controls (both Helmert contrasts were significant at p<0.01).

A two-way between-subjects ANCOVA was carried out to examine the effects of type and waveform on the eye velocity of the fellow eye under fellow eye viewing condition while controlling for visual acuity and stereopsis. There was a statistically significant main effect of type (F=83.3, p<0.01) and waveform (F=165.3, p<0.01) on the velocity of the fellow eye. In addition, significant effects of the covariates, visual acuity (F=120.9, p<0.01) and stereopsis (F=4.1, p=0.04) on the velocity of the fellow eye were observed. There was a statistically significant main effect of waveform (F=31.4, p<0.01) and type (F=3.2, p=0.07) on the velocity of the amblyopic eye while controlling for visual acuity and stereopsis. In addition, there was a significant effect of covariate stereopsis (F=58.2, p<0.01) but not the visual acuity (F=2.8, p=0.08) on the eye velocity of the amblyopic eye.

### Disconjugacy of Microsaccades and Quick Phases of Nystagmus

**Figure 6.**
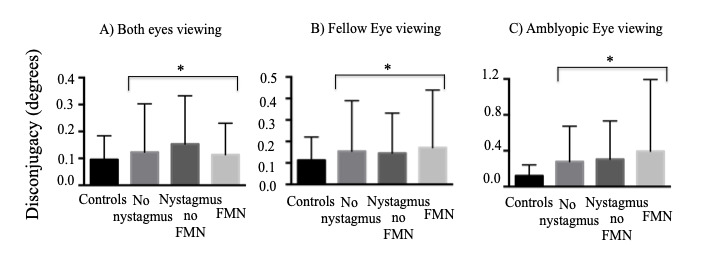
Mean and standard deviation of disconjugacy of composite amplitude of fixational saccades in controls and patients without nystagmus, and quick phases in patients with nystagmus across eye movement waveforms during both eyes viewing (A), fellow eye viewing (B) and amblyopic eye viewing (C) conditions. Amblyopic patients have increased disconjugacy under all viewing conditions. *=p<0.05, one way ANOVA

We have shown that patients with medium and large angle strabismus have an increase in the disconjugacy of fixational saccades in patients without nystagmus and quick phase of patients with nystagmus ( 23 ). In the current paper, we wanted to investigate the effects of presence of microstrabismus, severity of amblyopia and binocular function deficits on binocular coordination of the fixation eye movements. Thus, we analyzed the disconjugacy (difference in amplitude of the two eyes) of the fixational saccades in patients without nystagmus and quick phases in patients with nystagmus (Figure 6).

### Both eyes viewing

We found an increase in the disconjugacy of amplitude of fixational saccades in patients without nystagmus and quick phases in patients with nystagmus compared to controls (controls: 0.09° ± 0.08°, no nystagmus: 0.12° ± 0.17°, nystagmus no FMN: 0.15° ± 0.17°, FMN: 0.11° ± 0.11°; F=7.8, p<0.01) during both eyes viewing conditions (Fig 6A). A planned contrast analysis identified differences between controls and patients without nystagmus (p=0.01), controls and patients with nystagmus no FMN (p<0.01), and control and patients with FMN (p=0.02). No differences were seen between patients without nystagmus and patients with nystagmus (p=0.39). There was a difference with quick phases of patients with FMN being more disconjugate compared to patients with nystagmus without FMN (p=0.04).

We also evaluated the disconjugacy as a function of type of amblyopia during both eyes viewing conditions. The disconjugacy of fixational saccades and quick phases of nystagmus was greater in both anisometropic and mixed/strabismic amblyopic patients compared to controls (controls: 0.09° ± 0.08°, anisometropic amblyopia: 0.13° ± 0.21°, mixed/strabismic amblyopia: 0.12° ± 0.13°; F=10.1, p<0.01). A planned contrast analysis identified differences between controls and patients with anisometropia (p=0.01) and controls and patients with mixed/strabismic amblyopia (p<0.01). No difference in disconjugacy was observed between anisometropic versus mixed/strabismic amblyopia in the fellow eye (p=0.95) and amblyopic eye (p=0.43). A two-way between-subjects ANCOVA was carried out to examine the effects of type and waveform on the disconjugacy obtained under both eyes viewing conditions while controlling for visual acuity and stereopsis. No statistically significant differences were seen between type (p=0.3) and waveform (p=0.06) on the disconjugacy.

### Fellow Eye Viewing

When comparing the composite disconjugacy during fellow eye viewing condition (Fig 6B), the disconjugacy was greater in amblyopic patients across all eye movement waveforms compared to controls (controls: 0.11° ± 0.10°, no nystagmus: 0.15° ± 0.23°, nystagmus no FMN: 0.14° ± 0.18°, FMN: 0.17° ± 0.26°; F=16.3, p<0.01). A planned contrast analysis identified differences between controls and patients without nystagmus (p<0.01), controls and patients with nystagmus no FMN (p<0.01) and controls versus patients with FMN (p<0.01). No differences were observed between patients without nystagmus versus those with nystagmus (p=0.70). Patients with FMN had greater disconjugacy during fellow eye viewing condition than patients with nystagmus no FMN.

When comparing the composite disconjugacy across type of amblyopia, the disconjugacy was greater for anisometropic and strabismic/mixed amblyopia patients compared to controls (controls: 0.11° ± 0.10°, anisometropic amblyopia: 0.13° ± 0.20°, mixed/strabismic amblyopia: 0.16° ± 0.24°; F=27.0, p<0.01). A planned contrast analysis identified differences between controls and anisometropic amblyopia (p=0.03), controls and mixed/strabismic amblyopia (p<0.01) and anisometropic and mixed/strabismic amblyopia (p<0.01).

A two-way between-subjects ANCOVA was carried out to examine the effects of type and waveform on disconjugacy obtained during fellow eye viewing condition. There was a statistically significant effect of type of amblyopia (F=4.8, p=0.01) but not the waveform (F=1.7, p=0.17) while controlling for visual acuity and stereopsis. As predicted per our hypothesis, there was a significant effect of the covariate stereopsis (F=8.5, p<0.01) but not visual acuity of the fellow eye (F=0.5, p=0.46).

### Amblyopic Eye Viewing

When comparing the composite disconjugacy during amblyopic eye viewing condition (Fig 6C), the disconjugacy was greater in amblyopic patients across all eye movement waveforms compared to controls (controls: 0.12° ± 0.12°, no nystagmus: 0.27° ± 0.39°, nystagmus no FMN: 0.29° ± 0.40°, FMN: 0.39° ± 0.80°; F=68.3, p<0.01). A planned contrast analysis identified differences between controls and patients without nystagmus, controls and patients with nystagmus no FMN and controls versus patients with FMN. Significant differences were observed between patients without nystagmus versus those with nystagmus and patients with nystagmus with and without FMN (Helmert contrasts were significant at p<0.01).

When comparing the composite disconjugacy across type of amblyopia, the disconjugacy was greater for anisometropic and strabismic/mixed amblyopia patients compared to controls (controls: 0.12° ± 0.12°, anisometropia: 0.19° ± 0.32°, mixed/strabismic: 0.36° ± 0.63°; F=98.2, p<0.01). A planned contrast analysis identified differences between controls and anisometropic amblyopia, controls and mixed/strabismic amblyopia, and anisometropic and mixed/strabismic amblyopia (Helmert contrasts were significant at p<0.01).

A two-way between-subjects ANCOVA was carried out to examine the effects of type and waveform on disconjugacy obtained under amblyopic eye viewing condition. There was a statistically significant effect of waveform of fixation eye movements (F=5.6, p<0.01) and type of amblyopia (F=25.0, p<0.01) while controlling for visual acuity and stereopsis. In addition, the covariates, stereopsis (F=42.2, p<0.01) and visual acuity of the amblyopic eye (F=6.3, p=0.01) showed significant effects.

### Correlation between drift velocity and fixational saccade amplitude in controls and patients without nystagmus and quick and slow phase in patients with nystagmus

A Spearman correlation was performed between drift velocity and subsequent fixational saccade amplitude of the corresponding eye in patients without nystagmus and slow phase followed by the subsequent quick phase in patients with nystagmus. The greatest correlation coefficient (Spearman’s rho) values were seen under AEV, followed by FEV and then BEV in patients with FMN followed by nystagmus no FMN. In patients without nystagmus, the correlation coefficients were greater during AEV and BEV and least during FEV. Nevertheless, in all amblyopes this correlation between slow and fast eye movements was quite robust. A similar but much weaker correlation was also seen in control subjects (Table 3). We also explored a similar correlation as a function of type of amblyopia (Table 4). The strabismic amblyopes had greatest correlation followed by mixed and then anisometropic amblyopia (Tables 4).

**Table 3:** Correlation between inter-saccadic drift velocity/slow phase velocity and fixational saccade/quick phase nystagmus amplitude respectively by eye movement waveform

	Controls	No nystagmus	Nystagmus no FMN	FMN
Both eyes viewing	0.07, p=0.08 (0.08, p=0.06)	0.23, p<0.01 (0.04, p=0.06)	0.14, p=0.03 (0.28, p<0.01)	0.24, p<0.01 (0.27, p<0.01)
Fellow Eye viewing	0.08, p=0.06 (0.04, p=0.3)	0.12, p<0.01 (0.12, p<0.01)	0.28, p<0.01 (0.19, p<0.01)	0.80, p<0.01 (0.82, p<0.01)
Amblyopic Eye viewing	0.08, p=0.06 (0.04, p=0.3)	0.25, p<0.01 (0.17, p<0.01)	0.33, p<0.01 (0.28, p<0.01)	0.84, p<0.01 (0.85, p<0.01)

Both eyes viewing: top row: fellow eye, bottom row with parenthesis = amblyopic eye; Fellow and Amblyopic eye viewing: top row: viewing eye, bottom row: non-viewing eye; First value in each cell is the rho and second is the p value obtained by performing Spearman correlation.

**Table 4:** Correlation between inter-saccadic drift velocity/slow phase velocity and fixational saccade/quick phase nystagmus amplitude respectively by type

	Controls	Anisometropic	Mixed	Strabismic
Both eyes viewing	0.07, p=0.08 (0.08, p=0.06)	0.10, p=0.09 (0.06, p=0.33)	0.15, p<0.01 (0.15, p<0.01)	0.26, p=0.01 (0.17, p=0.02)
Fellow Eye viewing	0.08, p=0.06 (0.04, p=0.30)	0.10, p=0.01 (0.07, p=0.06)	0.36, p<0.01 (0.34, p<0.01)	0.72, p<0.01 (0.79, p<0.01)
Amblyopic Eye viewing	0.08, p=0.06 (0.04, p=0.30)	0.13, p<0.01 (0.11, p=0.01)	0.17, p<0.01 (0.16, p<0.01)	0.66, p<0.01 (0.71, p<0.01)

Both eyes viewing: top row: fellow eye, bottom row with parenthesis = amblyopic eye; Fellow and Amblyopic eye viewing: top row: viewing eye, bottom row: non-viewing eye; First value in each cell is the rho and second is the p value obtained by performing Spearman correlation.

## Discussion

The purpose of this study was to study the fixational instability in amblyopia patients using the waveform characteristics. We identified three fixation eye movement waveforms 1) patients with no nystagmus 2) patients with fusion maldevelopment nystagmus 3) patients with nystagmus but without the signature oculomotor markers of fusion maldevelopment nystagmus namely nasally directed slow phase under monocular viewing conditions and lack of reversal of direction of quick phase depending on the viewing eye. Our long-term goal is to identify biomarkers that can be used for screening as well as help predict treatment effectiveness and prognosis in amblyopia.

### BCEA as a measure of fixation stability:

BCEA has been increasingly used as a measure of fixational stability in amblyopic patients ( 23 , 25, 26, 27 ). In accordance with these previous studies, we also found that the BCEA measure reflects the fixation instability in amblyopic patients and accurately differentiates them from control subjects. However, BCEA takes into account all eye movements measured during the fixation period. We found that despite having nystagmus, the BCEA values were comparable or better during fellow eye viewing condition in patients with nystagmus versus those without nystagmus. This could be because the stereotyped, back-and-forth eye movements seen in nystagmus produces less overall scatter and results in a lower BCEA value. In other words, BCEA alone may underrepresent fixational instability in patients with nystagmus or FMN. We also found that BCEA measures were not able to identify differences across clinical amblyopia types, similar to prior reports ( 27 ). We computed vergence BCEA values (right eye position minus left eye position) obtained under both eyes viewing condition and found it to be greater in amblyopic patients compared to controls. However, similar to regular BCEA, vergence BCEA did not identify differences across eye movement waveforms or the clinical type of amblyopia. Thus, this highlights the importance of systematic evaluation of the waveforms of eye movements to precisely characterize the fixational instability in amblyopia patients.

We did not find any correlation between regular and vergence BCEA and visual acuity of the amblyopic eye. It is known that amblyopic patients without nystagmus have reduced microsaccade frequency with increased amplitude of fixational saccades, which correlates with the severity of amblyopia ( 23 , 24 ). We have also found increased eye position variance and eye velocities during inter-saccadic drifts, which are most pronounced in patients with strabismus with poor stereopsis and severe amblyopia patients ( 23 , 34 ). We have also found that amblyopic and strabismic patients with FMN have increased slow phase velocities compared to the inter-saccadic drifts in patients without nystagmus ( 16 , 34 ). BCEA does not differentiate and quantify fixational saccade and quick phase amplitudes and inter-saccadic drifts and slow phase velocities. BCEA also does not take into account the velocities of the eyes during fixation, an important parameter that can have effects on visual functions ( 16 ). Thus, it is important to study eye movement parameters such as amplitude of fast eye movements (fixational saccades and quick phases) and slow eye movements (eye position variance and eye velocities of inter-saccadic drifts and slow phase of nystagmus). Thus, to better understand the impact of type of amblyopia and presence of nystagmus on fixation stability, it is important to categorize fixational eye movements in amblyopia patients based on waveform, and parse the data into fast and slow eye movements.

### Fixational eye movements in the fellow and amblyopic eye during fellow, amblyopic and both eyes viewing conditions

We found that BCEA values are significantly greater in amblyopic patients compared to healthy controls under both eyes, fellow eye viewing and amblyopic eye viewing conditions. The instability as quantified by the BCEA measures were greater in the amblyopic eye compared to the fellow eye. This instability could arise due to an increase in the amplitude of fast eye movements and increase in the eye position variance and eye velocities during slow eye movements. Thus, as expected we found an increase in the amplitude of the fixational saccades and quick phases in both the fellow and amblyopic eye during fellow and amblyopic eye viewing conditions. We found a similar increase in the eye position variance and eye velocities of both the fellow and amblyopic eye compared to controls.

The fixation stability was better under both eyes viewing condition than monocular viewing conditions in normal subjects as measured by BCEA. This was also reflected by smaller fixational saccade/quick phase amplitude and disconjugacy and smaller eye position variance and lower drift velocity. These findings are consistent with previous studies that have shown that fixational stability is better under binocular viewing conditions than monocular ( 26 , 37 ). Fixation instability has been reported under binocular viewing conditions in the amblyopic eye of anisometropic and strabismic amblyopia patients ( 17 ). We found that the composite amplitude, eye position variance and eye velocities were greater in both the fellow and amblyopic eye compared to controls during both eyes viewing condition.

Besides these parameters, we also looked at the disconjugacy of fixational saccades in controls and patients without nystagmus and the quick phases of patients with nystagmus, defined as the absolute difference in composite amplitude between the two eyes. Earlier studies of microsaccades in normal subjects have showed them to be highly correlated, with disconjugacy in amplitude acting to correct disparities and reduce errors over time ( 42, 43, 44 ). We hypothesized that coordination of binocular vision is disrupted in amblyopia, which will then lead to greater disconjugacy, and that disconjugacy of fast eye movements is a reflection of fixational instability under binocular viewing conditions in amblyopic patients. It would also suggest that the vergence is unstable in amblyopic patients and that the disconjugacy could be a measure of a microstrabismus as some studies have shown ( 45 ). We found that, unsurprisingly, disconjugacy is increased in amblyopia, in both monocular and binocular eye viewing conditions with the greatest increase under amblyopic eye viewing condition. Thus, we have found several eye movement abnormalities in the fellow and amblyopic eye evident under both eyes viewing condition that could contribute to difficulties with reading and visuomotor task performance as has been reported in amblyopia patients ( 46 , 47 ).

### Fixational Eye Movements as a function of waveform and type of amblyopia

BCEA measures did not accurately distinguish patients with nystagmus from those without nystagmus. It also does not categorize patients based on the type of amblyopia. We found that the composite amplitude of fixational saccades and quick phases were greater in amblyopic patients. This increase in amplitude corresponds to the eye movement waveforms, with the greatest increase in FMN patients during fellow and amblyopic eye viewing conditions. We also found an increase in eye position variance and eye velocity in amblyopic patients, with the highest values in FMN patients. This is expected, as there is an increase in the nystagmus under monocular viewing conditions in FMN patients. On the other hand, the increase in amplitude was similar in the fellow and amblyopic eye of patients with and without nystagmus during both eyes viewing condition. This is likely due to a combination of increased amplitude of fixational saccades in amblyopic patients without nystagmus in conjunction with reduced nystagmus intensity in FMN patients under binocular viewing conditions. We also found a greater increase in the eye position variance and eye velocities in the amblyopic eye of patients with and without nystagmus compared to the fellow eye under both eyes viewing and monocular viewing conditions. Again, the increase in these parameters was greatest in FMN patients.

We also evaluated these parameters as a function of clinical type of amblyopia across all three viewing conditions. During both eyes viewing conditions, there was a similar increase in the amplitude of both the fellow and amblyopic eye of patients with anisometropic and mixed/strabismic amblyopia compared to controls. On the other hand, during fellow and amblyopic eye viewing condition, there was a greater increase in the amplitude of fellow and amblyopic eye of patients with mixed/strabismic amblyopia compared to anisometropic amblyopia. A similar trend was seen in the eye position variance and eye velocity increase under both eyes viewing and monocular viewing conditions. This suggests that presence of strabismus has greater deleterious effects on fixation stability during monocular viewing conditions irrespective of the eye movement waveform. We also found an increase in disconjugacy in patients with mixed/strabismic amblyopia compared to controls. This is expected as it has been shown previously that the presence of strabismus results in increased disconjugacy of fixational saccades/quick phases of nystagmus ( 29 , 34 ). We also found that anisometropic amblyopia patients had increased disconjugacy during both eyes viewing, fellow and amblyopic eye viewing conditions. Since they had greater disconjugacy compared to controls, this could possibly represent a microstrabismus. This suggests that the three different types of amblyopia are a spectrum rather than distinct categories. Alternatively, amblyopia alone, strabismus alone or both amblyopia and strabismus can disrupt the binocular co-ordination of fixational eye movements resulting in the increase in disconjugacy. In the future, a larger cohort of patients will allow us to independently analyze the effects of eye movement waveforms within each clinical subtype of amblyopia as well as delineate the effects of severity of amblyopia on the disconjugacy of fixational eye movements.

We also evaluated the disconjugacy of fast eye movements in patients separated by eye movement waveforms. We found that patients with nystagmus without FMN had the greatest disconjugacy under binocular viewing conditions compared to FMN patients. On the other hand, under monocular viewing conditions, the disconjugacy was greatest in patients with FMN. One potential explanation for this increased disconjugacy in patients without FMN is that microsaccades/fixational saccades are acting as a corrective measure for inter-saccadic drifts. Early studies of drifts suggested that, like microsaccades, they corrected fixational disparity ( 48 , 49 ). Thus, in normal vision, both microsaccades and drifts work to correct binocular disparities and maintain visual fusion. However, whereas microsaccades are so brief that they are not controlled with visual feedback ( 50 ), drifts depend on visual feedback for control. Because of this, it follows that regulation of drifts would be impaired in amblyopia where visual acuity is affected. Thus, the next question is whether these fixational saccades/quick phases are acting as a corrective measure for increased drift velocity. As outlined above, we found that patients with FMN and strabismus had higher drift velocities and greater increase in the microsaccade or quick phase amplitude. When we looked at the correlation between drift velocity and amplitude of the subsequent microsaccade/quick phase, we found that whereas there was almost no correlation in controls, there was a strong correlation for those subjects with FMN and strabismus. This supports our proposition that increased quick phase amplitude of nystagmus in amblyopes acts as a corrective measure for the increased slow phase drift velocity. This correlation was less marked in amblyopic patients with no nystagmus, suggesting that the increased amplitude and increased inter-saccadic drift are both secondary to amblyopia as opposed to the fixational saccades serving as a compensatory mechanism for the increased drift ( 23 ). This is further supported by the fact that the increase in amplitude was seen primarily under amblyopic eye viewing conditions in conjunction with increased disconjugacy of the fixational saccades.

### Effects of visual acuity and stereopsis on fixational eye movements

It remains controversial what role visual acuity plays in contributing to fixational instability. A recent study of fixational eye movements in amblyopes found that reducing the visual acuity of the fellow eye to match that of the amblyopic eye did not result in a loss of fixational stability, as measured by BCEA ( 51 ). These findings seem to suggest that reduced visual acuity is not responsible for a reduction in fixational stability. In the same study they reported that the higher BCEA values are related with worse visual acuity of the amblyopic eye. Subramanian et al. have found that visual acuity is correlated with worse BCEA in patients with strabismic but not anisometropic amblyopia ( 27 ). They also found that the fixation instability under binocular viewing condition as measured by vergence BCEA is related to stereopsis but not visual acuity. To date, very few studies have examined fixational saccades in amblyopia patients ( 25 , 26 , 51 ). Gonzalez et al. did not find any correlation between microsaccade amplitude and severity of amblyopia. However, they had only mild to moderate amblyopia patients in their cohort. On the other hand, multiple studies have shown that fixational saccade amplitude correlates with severity of amblyopia. Only one study to date has analyzed drifts in amblyopia patients and found that the drifts correlated with severity of amblyopia. They did not separate patients with nystagmus from those without nystagmus, a distinction that is necessary as outlined by our current paper. In addition, they measured the eye movements using a scanning laser ophthalmoscope and reported very high frequencies of fixational saccades (4-5 Hz). The authors have speculated that subtle head motion during the experiments could account for the increased frequency and possibly can also result in accurate drift assessments ( 25 ).

We evaluated fixational eye movement in subjects with corrected and uncorrected refractive errors and found a systematic increase in the amplitude of fixational saccades in the uncorrected state that correlated with the magnitude of the refractive error ( 52 ). We have also previously shown that the increase in fixational saccade amplitude correlates with severity of amblyopia. In the current paper, we found that both visual acuity of the amblyopic eye and stereopsis had effects on composite amplitude of fast eye movements of fellow and amblyopic eye under both eye viewing and amblyopic eye viewing conditions. Acuity of the amblyopic eye had a significant effect on eye position variance and stereopsis had a significant effect on eye velocity of the amblyopic eye under both eyes viewing condition. During fellow eye viewing, both fellow eye acuity and stereopsis had a significant effect on eye position variance and eye velocity of the fellow eye. On the other hand, under amblyopic eye viewing, stereopsis but not visual acuity of the amblyopic eye had effects on the eye velocity of the amblyopic eye. For disconjugacy, no effects of stereopsis or acuity of the amblyopic eye were seen when taking into account both the waveform and type of amblyopia. On the other hand, during fellow eye viewing, stereopsis but not the visual acuity of the fellow eye had an effect on disconjugacy, and during amblyopic eye viewing both stereopsis and visual acuity of the amblyopic eye had effects on the disconjugacy. These results suggest that amplitude of fast eye movements can reliably predict the severity of amblyopia. The presence of increased eye velocities of slow eye movements of the amblyopic eye can be used as a marker of stereopsis function.

## Conclusions

The findings from our current study highlight the need to characterize the fixational eye movements in amblyopia patients by their waveforms. This approach has allowed us to identify specific eye movement biomarkers that better predict the type and severity of amblyopia and are reflective of the measures of both visual acuity and stereopsis. Future studies would focus on using these eye movement waveforms and fast and slow fixational eye movements to assess visuomotor function deficits seen in amblyopia children. These parameters can also be used to predict prognosis of monocular and binocular amblyopia treatment.

## Ethics and Conflict of Interest

The authors declare that the contents of the article are in agreement with the ethics described in http://biblio.unibe.ch/portale/elibrary/BOP/jemr/ethics.html and that there is no conflict of interest regarding the publication of this paper.

## Acknowledgements

This work was supported by grants from Knights Templar Research Foundation (FG), and Fight for Sight Foundation (FG), Blind Children’s Center (FG), RPB Unrestricted grant CCLCM-CWRU and Dystonia Medical Research Foundation clinical fellowship award (AS). Sarah Kang was supported by a Summer Student Fellowship from the Parkinson’s Disease Foundation.
